# *UBA1-CDK16*: A female-specific chimeric RNA emerging through evolution and involved in immune regulation

**DOI:** 10.1126/sciadv.adz9784

**Published:** 2026-05-29

**Authors:** Xinrui Shi, Loryn Blackburn, Sandeep Singh, Martyna Glowczyk-Gluc, Anam Tajammal, Shafaque Zahra, Shailesh Kumar, Robert Cornelison, Chen Liang, Fujun Qin, Aiqun Liu, Shitong Lin, Yue Tang, Justin Elfman, Thomas Manley, Timothy Bullock, Doris M. Haverstick, Peng Wu, Hui Li

**Affiliations:** ^1^ Department of Biochemistry and Molecular Biology, School of Medicine, University of Virginia, Charlottesville, VA 22908, USA.; ^2^ Division of Human Genetics, Cincinnati Children’s Hospital Medical Center, Cincinnati, OH 45229, USA.; ^3^ Department of Pathology, School of Medicine, University of Virginia, Charlottesville, VA 22908, USA.; ^4^ National Institute of Plant Genome Research (NIPGR), New Delhi 110067, India.; ^5^ College of Animal Science and Veterinary Medicine, Shanxi Agriculture University, Jinzhong, Shanxi 030801, China.; ^6^ School of Basic Medical Sciences, Academy of Medical Sciences, Zhengzhou University, Zhengzhou 450001, China.; ^7^ Department of Endoscopy, Affiliated Tumor Hospital of Guangxi Medical University, Nanning, Guangxi 530021, China.; ^8^ Department of Obstetrics and Gynecology, Union Hospital, Tongji Medical College, Huazhong University of Science and Technology, Wuhan, Hubei 430030, China.; ^9^ Cancer Biology Research Center (Key Laboratory of the Ministry of Education), Tongji Hospital, Tongji Medical College, Huazhong University of Science and Technology, Wuhan, Hubei 430030, China.; ^10^ Ascentage Pharma Group Inc., Rockville, MD 20850, USA.; ^11^ Perelman School of Medicine, University of Pennsylvania, Philadelphia, PA 19104, USA.; ^12^ Center for RNA Science and Medicine, University of Virginia, Charlottesville, VA 22908, USA.

## Abstract

Chimeric RNAs resulting from intergenic splicing represent a distinct mechanism for transcriptome expansion. To explore the role of this previously unidentified layer of the transcriptome in sex-specific immunity, we analyzed RNA sequencing data from 425 blood samples and identified a female-specific chimeric RNA, *UBA1-CDK16*, which was further validated in more than 1200 blood samples. This chimeric RNA forms via cis-splicing between two adjacent X-linked parental genes, *UBA1* and *CDK16*, despite both being expressed in both sexes. We demonstrated that a female-specific chromatin loop at the *UBA1-CDK16* junction sites facilitates the intergenic splicing. Evolutionary analysis revealed that *UBA1-CDK16* became female specific in humans through at least two independent paths. Functional studies suggested that *UBA1-CDK16* is enriched in the myeloid lineage and may regulate myeloid cell development. Notably, its abnormal expression in female patients with COVID-19 correlates with altered neutrophil counts, highlighting its potential role in the disease progression.

## Teaser

A female-specific chimeric RNA driven through chromatin looping may influence immunity and blood cell development.

## INTRODUCTION

The transcriptomic complexity is not completely represented by the genome size and the number of genes (*1*). Alternative splicing (*2*), along with other processes such as RNA editing (*3*) and alternative polyadenylation (*4*), is known to expand the functional genome. Chimeric RNAs are fusion transcripts composed of sequences transcribed from disparate parental genes (*5*, *6*). Traditionally, chimeric RNAs were considered products of chromosomal rearrangement in cancer cells (*7*–*10*). However, growing evidence showed that many chimeric RNAs arise from intergenic splicing and have been identified in noncancerous cells and tissues (*11*–*13*), suggesting their role in diversifying the transcriptome (*5*, *14*). These mechanisms include trans-splicing (*11*), involving the fusion of exons from different transcripts, and transcriptional read-through [also known as cis-splicing between adjacent genes (cis-SAGe)] (*15*, *16*), which occurs when the transcriptional machinery continues beyond the typical gene termination site and enters the adjacent gene. Although the landscape of different types of chimeric RNAs has been profiled across various diseases (*17*–*20*), their significance is less appreciated because of limited understanding of their molecular origins and functions.

Sexual dimorphism in biology has been shaped through evolution and is influenced by multiple factors, including sex chromosomes, sex hormones, transcriptomic differences, and epigenetic mechanisms. In humans, these differences are notably pronounced in the immune system, leading to distinct immune responses and disease susceptibilities (*21*). Studies have shown that females often mount stronger immune responses to infections than males and tend to have higher prevalence of autoimmune diseases (*22*, *23*). A key contributor to sex-biased immunity is the sex chromosome. Several immune-related genes and components of the nuclear factor κB pathway are located on the X chromosome. In some cases, these genes escape X chromosome inactivation, leading to elevated expression levels that have been linked to increased autoimmune risk (*24*–*26*). In addition, epigenetic processes such as X-inactivation skewing have been implicated in modulating immune responses and autoimmune disease susceptibility (*27*). Recent work has further highlighted the role of *XIST* and its associated regulatory complexes in shaping sex-specific gene expression within immune cells (*28*–*30*). Beyond sex chromosome-linked effects, transcriptomic differences between males and females represent another crucial but less understood layer of immune regulation. Emerging studies have begun to profile sex-specific gene expression patterns across immune cells, revealing widespread transcriptomic variation (*31*, *32*). However, the molecular basis of sex-biased immunity and differential disease susceptibility remains incompletely understood. In this study, we aim to expand our understanding of sex-specific transcriptomic regulation in the human immune system by uncovering an unrecognized female-specific chimeric RNA from whole blood samples spanning diverse immune cell types.

Here, we investigate sex-biased chimeric RNAs in blood cells and report an X-linked, female-specific chimeric RNA. We comprehensively study the mechanism underlying its female-specific intergenic splicing, the evolutionary conservation of its splice sites, and its functional role in myeloid development, inflammatory responses, and the response to COVID-19, with potential relevance to other infectious diseases. These findings support the notion that chimeric RNAs constitute a distinct class of transcripts, regulated independently of their parental genes, and represent a distinct layer of RNA variation with potential implications for sex differences in immune responses and susceptibility to infectious diseases.

## RESULTS

### Identification of a female-specific *UBA1-CDK16* chimeric RNA in human blood

**Fig. 1.**
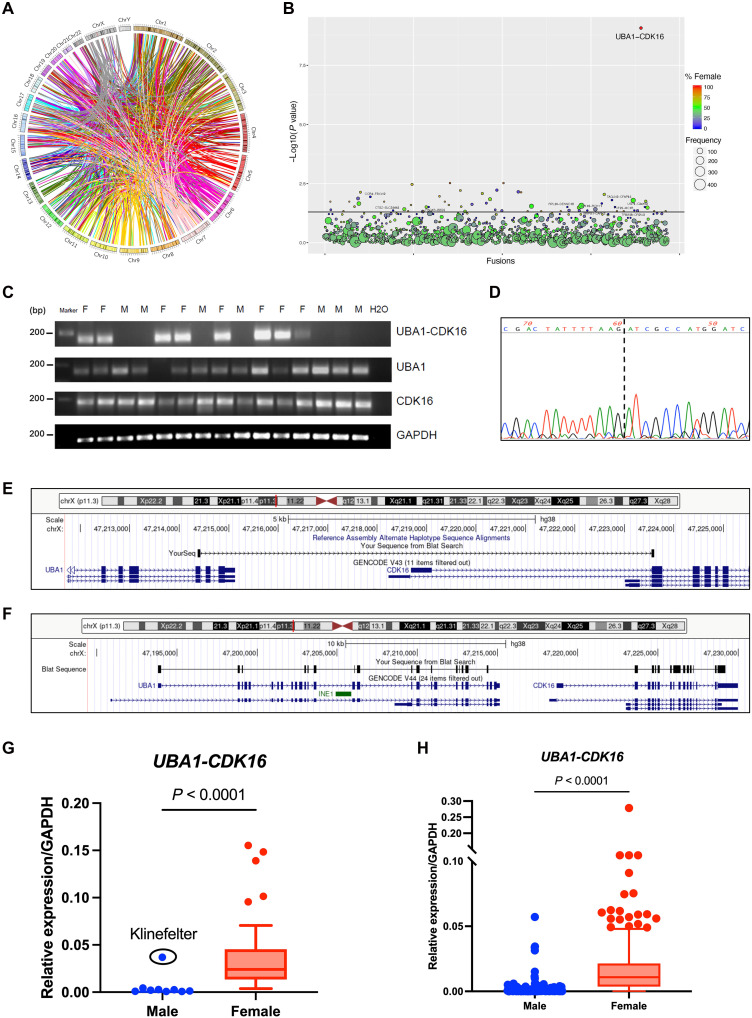
Finding and validation of *UBA1-CDK16* chimeric RNA. (**A**) Circos plot depicted chimeric RNAs found across the genome from 425–paired-end RNA-seq data of GTEx whole blood. A total of 1156 recurrent fusion events (≥5) are shown, with “MM” fusions excluded. Lines denote the chimeric RNAs connecting two parental genes. (**B**) Bubble plot highlighting the significant sex-bias of *UBA1-CDK16* chimeric RNA. The size of each bubble indicates the number of samples with positive detection of candidate chimeric RNAs, while the color scale indicates the percentage of female samples that contain a particular chimera. The *x* axis plots the distribution of chimeric RNAs. The *y* axis plots the negative value of the log (base10)–transformed *P* value obtained via chi-squared tests. (**C**) RT-PCR validation of *UBA1-CDK16* chimeric RNA and parental genes in 15 whole blood samples from eight female (F) and seven male (M) healthy individuals. Primers flanking the predicted fusion junction site were used for PCR amplification from cDNA. Gel electrophoresis revealed the presence of the chimeric RNA in female samples, and absence in male samples. Primers specific for the parental genes were used to detect *UBA1* and *CDK16* transcripts, together with primers for the control gene *GAPDH*. (**D**) Junction sequence of the RT-PCR product of the chimeric RNA obtained by Sanger sequencing. (**E**) Configuration of the Sanger-sequenced part of chimeric RNA is depicted on the UCSC Genome Browser (hg38). (**F**) Configuration of the full-length sequence of chimeric RNA is depicted on the UCSC Genome Browser (hg38). (**G**) RT-qPCR analysis of the *UBA1-CDK16* chimeric transcript in 104 RNA samples obtained from GTEx, including 64 males and 40 females. Expression was normalized to that of *GAPDH*. (**H**) RT-qPCR analysis of *UBA1-CDK16* chimeric transcript in 1252 clinical samples, including 727 males and 525 females. Expression was normalized to that of *GAPDH*.

Chimeric RNAs were identified using EricScript (*33*), a computational tool that detects candidate fusion events from paired-end RNA sequencing (RNA-seq) data on the basis of discordant reads and junction-spanning reads. We first examined the chimeric RNA transcriptome of whole blood samples within Genotype-Tissue Expression (GTEx) database across healthy individuals (*34*, *35*). A total of 57,063 chimeric RNAs were identified from the 425–whole blood RNA-seq dataset in GTEx, from 157 women and 268 men. Chimeric RNAs were classified on the basis of the junction site location: If the junction site fell within 2 bp of the canonical exon boundary, the junction was annotated as “E” (edge of exon); otherwise, it was annotated as “M” (middle of exon). To reduce potential false positives identified by the software, we filtered out “M/M” fusions (*36*) and focused on recurrent chimeric RNAs found in more than five individuals, which resulted in the final selection of 1156 chimeric RNAs (Fig. 1A; fig. S1, A and B; and table S1).

With the aim to identify sex-specific chimeric RNAs, we compared the distribution of recurrent chimeric RNAs between sexes. Notably, we found a chimeric transcript, *UBA1-CDK16*, which exhibited substantial bias toward female expression (24 of 157 in females and 0 of 268 in males) (Fig. 1B). Bioinformatic detection of *UBA1-CDK16* across multiple female tissues in GTEx RNA-seq data indicated that its expression is largely restricted to blood (fig. S1C). Recognizing the sensitivity limitations of RNA-seq and bioinformatic detection, we conducted further validation of this female specificity through reverse transcription polymerase chain reaction (RT-PCR) on RNA extracted from 15 whole blood samples from healthy donors (eight females and seven males). PCR primers for *UBA1-CDK16* were designed at the predicted junction sites, while primers for each wild-type parental gene were designed at sequences not involved in chimeric RNA (table S2). Our experimental results confirmed the exclusive female expression pattern of *UBA1-CDK16*, while transcripts from the two parental genes *UBA1* and *CDK16* did not exhibit any sex-specific expression (Fig. 1C and fig. S2). Sanger sequencing of the chimeric PCR product confirmed the junction sequence (Fig. 1D), revealing a fusion of the third to the last exon (exon 24 based on RefSeq gene NM_003334) of *UBA1* with the second exon (exon 2 based on RefSeq gene NM_006201) of *CDK16*, with the breakpoints occurring at chrX:47,214,428 and chrX:47,223,552 in human genome (GRCh38/hg38) (Fig. 1E). The female specificity of the chimeric RNA suggests a specific mechanism contributing to this sex-biased chimeric transcript expression and a possible functional role different from parental genes. To predict the potential product of *UBA1-CDK16*, we conducted PacBio long-read sequencing in female peripheral blood mononuclear cells (PBMCs) to identify the full-length sequence of the chimeric RNA. The sequences of *UBA1-CDK16* beyond the junction site are distinct from the respective parental gene isoforms (Fig. 1F), resulting in an absence of extended open reading frames (fig. S3A). Our cell fractionation analysis also suggested that *UBA1-CDK16* is likely a long noncoding chimeric RNA, given its notable enrichment in the nucleus (fig. S3B).

To further validate the pronounced female expression bias of *UBA1-CDK16*, we conducted RT-quantitative PCR (RT-qPCR) analysis on an additional 104 whole blood RNA samples directly obtained from GTEx (fig. S4A). This expanded analysis revealed notable sex differences, as all female samples exhibited positive expression, while all male samples showed negative expression, except for a single outlier from a Klinefelter syndrome (47,XXY) donor (Fig. 1G). Our investigation found no correlation between the expression of *UBA1-CDK16* and factors such as age, body mass index (BMI), or population (fig. S4, B and C). Furthermore, we observed no differential expression of *UBA1-CDK16* across different menopausal status groups, indicating an independent regulation from sex hormones (fig. S4D). In summary, our findings from both bioinformatic identification and experimental validation have unveiled a *UBA1-CDK16* chimeric RNA that exhibits significant sex differences in expression among individuals with one (XY) or two X chromosomes (XX or XXY).

### Further validation of female specificity of *UBA1-CDK16* chimeric RNA in 1252 clinical blood samples

To further confirm the sex-biased expression of this chimeric RNA, we validated its expression in 1252 clinical blood samples, comprising 525 females and 727 males (fig. S5A). To correct for specimen labeling errors and potential transgender situations, we examined the expression of a Y-linked gene *DDX3Y*. In accordance with our previous findings, the majority of female samples exhibited positive *UBA1-CDK16* expression (474 of 525), while a small proportion of male samples also showed a positive detection of *UBA1-CDK16* (173 of 727). The sex-biased difference in *UBA1-CDK16* expression was found to be highly significant (*P* = 2.7 × 10^−53^) (Fig. 1H). We next analyzed the correlation between *UBA1-CDK16* and its parental genes in female clinical blood samples with detectable *UBA1-CDK16* expression and found no statistically significant association, further supporting the independent female-biased expression of this chimeric RNA (fig. S5B). Consistent with GTEx samples, our analysis revealed no statistically significant contributions of age, population, BMI, or menopausal status groups to the variation in *UBA1-CDK16* levels (fig. S5, C to E). The presence of outlier samples (females lose or males gain the chimeric RNA) in this clinical collection, but not in the healthy donors from GTEx, suggested that this sex-specific chimeric RNA may be affected by multiple factors including certain physiological conditions, disease-or treatment-associated effects, or other environmental influences.

### *UBA1-CDK16* is a product of cis-SAGe from the inactive X chromosome

**Fig. 2.**
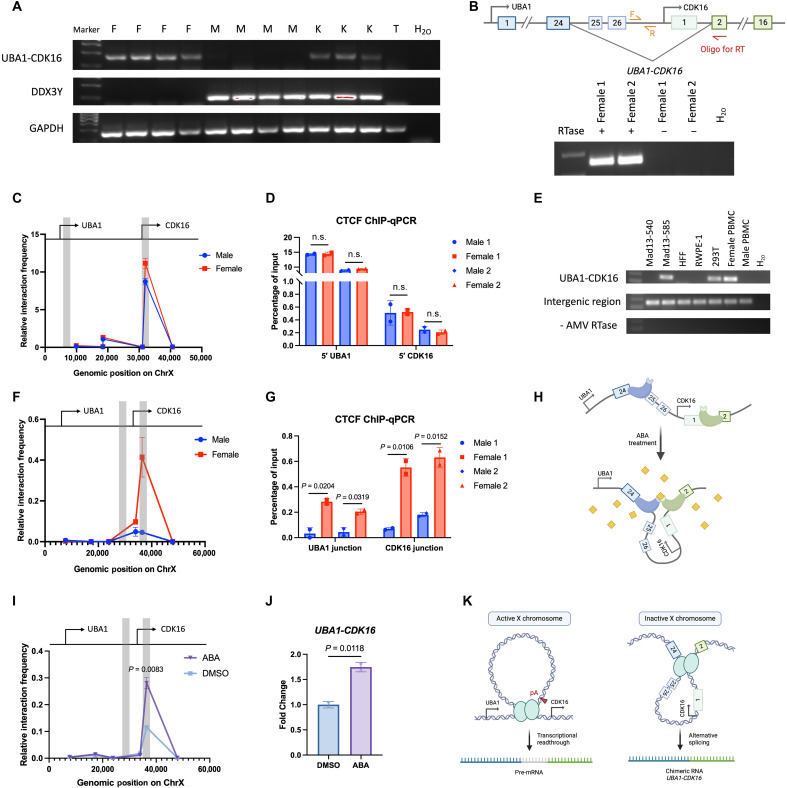
Mechanism of UBA1-CDK16 chimeric RNA expression from the inactive X chromosome. (**A**) RT-PCR detected *UBA1-CDK16* in females (F) and patients with Klinefelter syndrome (K), but not in males (M) or patients with Turner syndrome (T). DDX3Y confirmed Y chromosome presence in males and Klinefelter samples. *GAPDH* served as control. (**B**) Schematic of cis-SAGe PCR with a reverse transcription oligo annealing to *CDK16* exon 2 and PCR primers flanking the intergenic region. Cis-SAGe PCR products were detected in DNase I–treated female PBMCs. No RTase served as negative control. (**C**) 3C qPCR analysis of *UBA1-CDK16* locus interactions in male and female PBMCs using Bam HI, with 5′ *UBA1* and 5′ *CDK16* fragments as anchors (gray bars). (**D**) ChIP-qPCR demonstrated CTCF-binding at 5′ *UBA1* and 5′ *CDK16* in PBMCs, with no significant sex differences (*n* = 2). (**E**) RT-PCR revealed the mature *UBA1-CDK16* expression in female cell lines. Cis-SAGe PCR revealed the intergenic region detection in all cell lines. No RTase served as negative control. (**F**) 3C qPCR analysis of *UBA1-CDK16* locus interactions in male and female PBMCs using Bgl II, with *UBA1* and *CDK16* junction sites as anchors (gray bars). (**G**) ChIP-qPCR demonstrated significantly higher CTCF-binding at both junction sites in female PBMCs (*n* = 2). (**H**) Schematic of CLOuD9 system. ABA (yellow) induced dimerization of complementary constructs (blue and green) targeted by sgRNAs, inducing chromatin loop formed between junction sites. (**I**) 3C qPCR analysis in HEK293T cells after ABA treatment showed increased interaction between *UBA1* and *CDK16* junction sites (gray bars). DMSO, dimethyl sulfoxide. (**J**) RT-qPCR showed that CLOuD9-induced chromatin looping between junction sites increased *UBA1-CDK16* expression (*n* = 2). (**K**) Model of chromatin architecture at the *UBA1-CDK16* locus on active and inactive X chromosomes. Blue circle represents the CTCF protein. Black arrows represent the transcription start sites. Red arrow represents the poly-A site.

In female cells, random inactivation of one of the X chromosomes serves to compensate for the normal dosage differences of X chromosome DNA between females and males. Given the female-specific characteristics of the X-linked chimeric RNA, *UBA1-CDK16*, we suspected that it is produced from the inactive X chromosome. We then obtained clinical samples from several patients with Klinefelter syndrome and one patient with Turner syndrome. Klinefelter syndrome is a chromosomal disorder in males characterized by the presence of an additional X chromosome (47,XXY), while Turner syndrome is a rare chromosomal condition in females resulting from the complete or partial loss of one X chromosome, most commonly presenting as 45,X. *UBA1-CDK16* was detected in all patients with Klinefelter syndrome, while it was absent in the patient with Turner syndrome (Fig. 2A). This finding suggests that the *UBA1-CDK16* chimeric RNA is exclusively expressed from the inactive X chromosome.

*UBA1* and *CDK16* are neighboring genes located on the X chromosome. To investigate the mechanisms responsible for the formation of this chimeric RNA, we initially examined whole-genome sequencing data from GTEx and confirmed that it is not a product of an interstitial deletion (fig. S6A). Given that the GTEx whole-genome sequencing data typically achieve a median depth of approximately 32× and no interstitial deletions have been identified between the junction sites of *UBA1-CDK16*, the chimera is unlikely to be the result of heterozygous deletions in females. *UBA1* and *CDK16* are transcribed in the same orientation, fitting the characteristics of cis-SAGe. To further confirm that *UBA1-CDK16* is a product of cis-SAGe, we designed a cis-SAGe RT-PCR assay to detect the precursor read-through mRNA. In this assay, we used an oligo annealing to exon 2 of *CDK16* to specifically reverse-transcribe the precursor read-through mRNA. This read-through precursor was then successfully detected using intergenic primers in female PBMCs (Fig. 2B). To ensure the elimination of DNA contamination, RNA was treated with deoxyribonuclease I (DNase I), and the no-reverse transcriptase (no-RTase) control confirmed the complete removal of DNA contaminants.

### A 5′-5′ chromatin loop mediates sex-independent transcriptional read-through

Transcriptional read-through has been reported to be associated with repeat elements at transcription termination sites (*37*). However, we did not observe enrichment of repeat elements at either the transcription termination site of *UBA1* or the junction sites of *UBA1-CDK16* (fig. S6B). To delve further into the molecular mechanisms underlying the formation of *UBA1-CDK16* through cis-SAGe, we examined the inactive X chromosome (Xi) structure by comparing male and female PBMCs. Prior studies have reported that the intergenic chromatin loops formed between promoters can promote the transcriptional read-through by obstructing the poly-A recognition site (*38*). To investigate this, we conducted Chromosome Confirmation Capture (3C), which revealed a prominent chromatin loop between the 5′ regions of *UBA1* and *CDK16*, characterized by notable high interaction frequencies (Fig. 2C). Subsequently, we performed CCCTC-binding factor (CTCF) chromatin immunoprecipitation (ChIP) analysis and identified notable CTCF binding at the predicted CTCF-binding sites around the loop anchors (fig. S6, C and D). Two sites exhibiting the most robust and reproducible CTCF enrichment were selected for comparison of CTCF binding frequency in two pairs of male and female PBMCs. However, no sex differences were observed in the chromatin loop (Fig. 2C) or in CTCF binding at both 5′ regions of *UBA1* and *CDK16* (Fig. 2D).

To further investigate the role of this 5′-5′ chromatin loop in mediating *UBA1-CDK16* transcriptional read-through, we induced this chromatin loop in human embryonic kidney (HEK) 293T cells through CRISPR-mediated chromatin loop organized using dCas9 (CLOuD9) (*39*). HEK293T, known for its high transfection efficiency, is a female embryonic kidney cell line with low *UBA1-CDK16* expression (Fig. 2E). Using single guide RNAs (sgRNAs) targeting the identified loop anchor sites, we induced an in vitro chromatin loop through dCas9 dimerization, followed by treatment with abscisic acid (ABA) (fig. S6E). Facilitated by this chromatin loop, we observed a significant increase of transcriptional read-through precursors, as detected by cis-SAGe RT-PCR. Notably, this increase in read-through precursors did not affect the mature *UBA1-CDK16* expression (fig. S6F).

Given that the chromatin loop between 5′-5′ regions of *UBA1* and *CDK16* is detected in both female and male samples, we postulated that the transcriptional read-through precursors are present in both sexes. To test this hypothesis, we measured *UBA1-CDK16* and its precursor in seven different cell lines, encompassing both male and female cell lines. We observed the presence of read-through precursors in all cell lines, irrespective of gender (Fig. 2E). However, the mature chimeric transcript *UBA1-CDK16* was specifically produced in female cell lines, including primary female endometrial fibroblast cell line Mad13-585, female embryonic kidney cell line 293 T, and female PBMCs. These findings collectively highlight the sex-independent role of the 5′-5′ chromatin loop in mediating transcriptional read-through and suggest a potential female-specific alternative splicing event leading to the expression of *UBA1-CDK16*.

### A female-specific chromatin loop is formed between *UBA1-CDK16* junction sites

Considering the distinct female-specific expression characteristics of *UBA1-CDK16* and the presence of its read-through precursors in both sexes, we posited that a specific chromatin structure on the inactive X chromosome might contribute to the alternative splicing of *UBA1-CDK16* (*40*). To test this hypothesis, we compared chromatin interactions between the junction sites in male and female PBMCs using 3C. Our study identified a chromatin loop between the junction sites, with a higher level of interaction in female PBMCs (Fig. 2F). Notably, we also detected CTCF binding around the anchor sites (fig. S6, G and H). Two sites exhibiting the most robust and reproducible CTCF enrichment were selected for comparison of CTCF binding frequency between male and female PBMCs. In two pairs of donors, we observed significant female-specific CTCF binding at both junction sites (Fig. 2G).

To prove the role of this junction chromatin loop in *UBA1-CDK16* expression, we conducted CLOuD9 experiments with sgRNAs targeting the identified junction anchor sites (Fig. 2H). This manipulation induced a notable increase in chromatin interaction between the junction sites (Fig. 2I) and resulted in elevated *UBA1-CDK16* expression (Fig. 2J). Therefore, this female-specific chromatin loop, which brings the junction splicing sites into proximity, promotes the expression of the chimeric transcript *UBA1-CDK16*.

In summary, our findings highlight that while a chromatin loop formed between the 5′ regions of *UBA1* and *CDK16* mediates transcriptional read-through in both males and females, the female-specific intergenic chromatin loop at the junction site is the key to facilitate intergenic alternative splicing that generates *UBA1-CDK16* chimeric transcript on the inactive X chromosome (Fig. 2K).

### Appearance of *UBA1-CDK16* during evolution

**Fig. 3.**
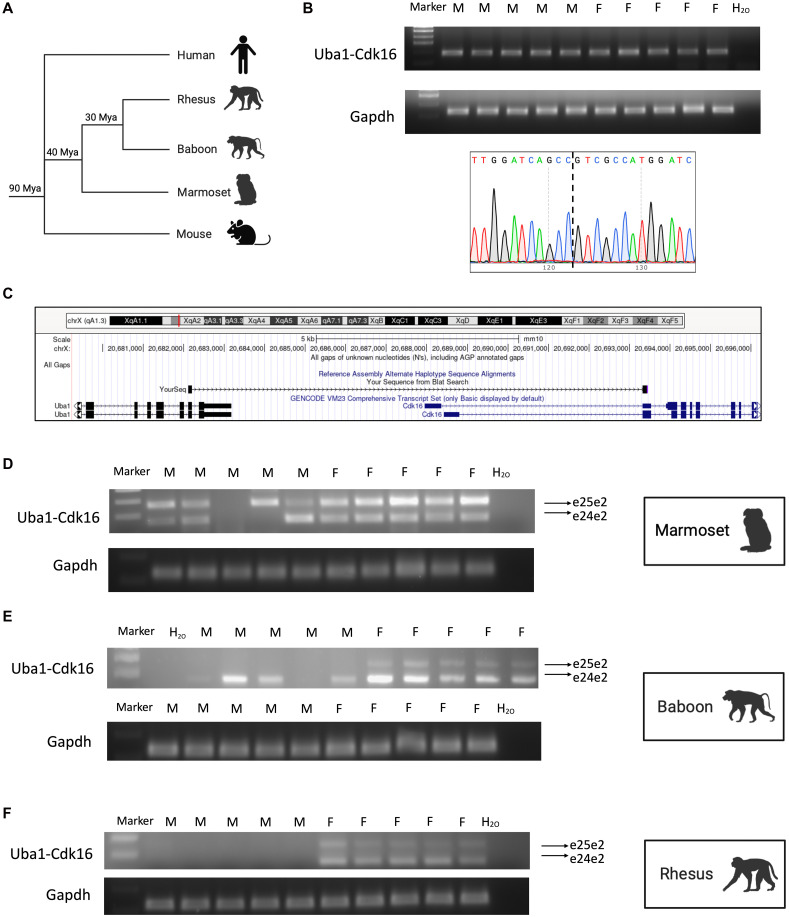
Comparative study of *UBA1-CDK16* expression during evolution. (**A**) The schematic phylogenies represent evolutionary relationships among mice, nonhuman primates, and humans. (**B**) Gel electrophoresis revealed the presence of the chimeric RNA *Uba1-Cdk16* in both male and female mice. RNA was extracted from buffy coat. The junction sequence of the RT-PCR product of the chimeric RNA obtained by Sanger sequencing is shown below. (**C**) Configuration of the Sanger-sequenced part of chimeric RNA is viewed on the UCSC Genome Browser (mm10). (**D** to **F**) Gel electrophoresis revealed the presence of different isoforms of the chimeric RNA *Uba1-Cdk16* in marmosets, baboons, and rhesus monkeys separately.

To explore the significance of the chimeric transcript *UBA1-CDK16* in blood, we first examined its presence in an evolutionary context by checking its expression across species from *Mus musculus* to *Homo sapiens* (Fig. 3A). Our findings unveiled the presence of *Uba1-Cdk16* in both male and female mice (Fig. 3B). However, Sanger sequencing of the chimeric PCR product indicated the existence of a distinct e25e2 isoform (exon 25 of *Uba1* and exon 2 of *Cdk16* based on the Refseq genes NM_009457 and NM_011049), different from the human e24e2 form (Fig. 3C). It has been reported that the most frequent architecture of read-through transcription involves a junction between the second to the last exon of the upstream gene and the second exon of the downstream gene, consistent with the e25e2 isoform (*41*). As we traced its evolutionary trajectory in nonhuman primates, we observed a notable gain of the e24e2 isoform in marmosets, but without a sex-biased expression pattern for either form (Fig. 3D). In baboons, the e25e2 isoform further evolved to become female specific, coexisting with e24e2 isoform in both sexes (Fig. 3E). In rhesus monkeys, both e24e2 and e25e2 isoforms exhibited female-specific expression (Fig. 3F). The presence of two isoforms, with e25e2 being female specific in baboons and both isoforms female specific in rhesus monkeys, suggests that two evolutionary paths converged to result in e24e2 as the exclusive isoform present in the human females. The emergence of the female-specific chimeric RNA *UBA1-CDK16* during this dynamic evolution process indicates its potential functional roles in the blood system.

### *UBA1-CDK16* inhibits myeloid differentiation

**Fig. 4.**
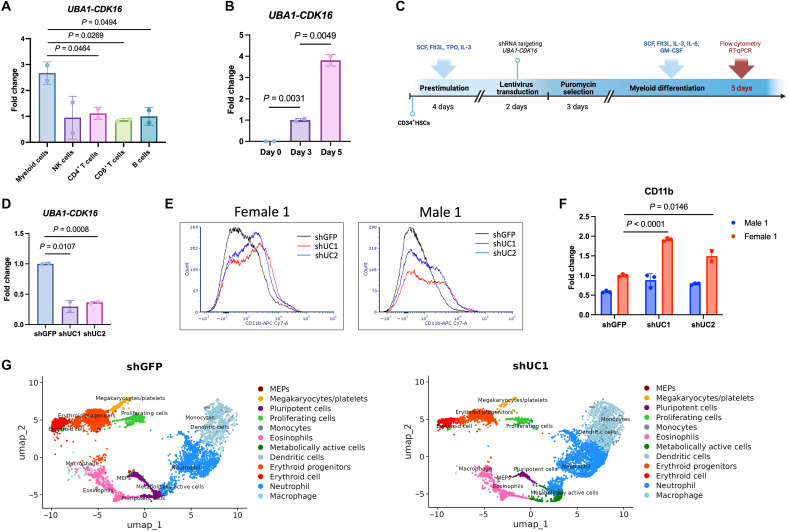
Function of *UBA1-CDK16* during myeloid differentiation. (**A**) RT-qPCR detection of chimeric RNA *UBA1-CDK16* in myeloid cells, NK cells, CD4^+^ T cells, CD8^+^ T cells, and B cells sorted from human PBMCs (*n* = 2). (**B**) RT-qPCR detection of chimeric RNA *UBA1-CDK16* on days 0, 3, and 5 of CD34^+^ cells myeloid differentiation (*n* = 2). (**C**) Experimental timeline for shRNA transduction of expanded CD34^+^ cells followed by myeloid differentiation. (**D**) The effect of *UBA1-CDK16* knockdown by shUC1 and shUC2. Short hairpin green fluorescent protein (shGFP) was used as negative control (*n* = 2). (**E**) The fluorescent intensity distribution of CD11b in CD34^+^ cells undergo myeloid differentiation upon *UBA1-CDK16* knockdown. Gated-out single cells were used for fluorescence-activated cell sorting (FACS) analysis. (**F**) *CD11b* mRNA expression detected by RT-qPCR. RNA samples were extracted from day 5 of myeloid differentiated cells. (*n* = 3). (**G**) Uniform manifold approximation and projection (UMAP) projection of single-cell transcriptomes from shUC1 and shGFP samples from a female donor, clustered into major blood cell types. Cell types were annotated on the basis of canonical marker gene expression. MEPs, megakaryocyte-erythroid progenitors.

*UBA1-CDK16* was found as a female-specific chimeric RNA preferentially present in peripheral blood, promoting the hypothesis that it may be involved in immune processes, given that white blood cells are the key component of immune responses. To investigate the potential function of *UBA1-CDK16* in the blood immune system, we sorted specific blood cell subsets [CD4^+^ T cells, CD8^+^ T cells, CD19^+^ B cells, CD56^+^ natural killer cells (NK cells), and CD11b^+^ myeloid cells] from normal female PBMCs as described in Materials and Methods (“Cell sorting” section) and examined the expression of *UBA1-CDK16* (*42*). Our findings revealed a significant enrichment of *UBA1-CDK16* in myeloid lineage cells compared to its expression in lymphoid lineage cells including NK cells, T cells, and B cells (Fig. 4A). To gain deeper insight into the dynamic expression of *UBA1-CDK16* during the myeloid lineage commitment, we initially investigated its presence in hematopoietic stem cells (HSCs). Unexpectedly, this chimeric RNA is undetectable in CD34^+^ HSCs isolated from peripheral blood. However, both parental transcripts, *UBA1* and *CDK16*, were expressed in both CD34^+^ and CD34^−^ cells at similar levels (fig. S7A). This finding suggested that the formation mechanism and potential function of *UBA1-CDK16* are likely to be differentiation associated rather than an early stem cell feature. We then induced the differentiation of HSCs into the myeloid lineage by treating them with a combination of cytokines, including stem cell factor (SCF), FMS-like tyrosine kinase 3 ligand (Flt3L), interleukin-3 (IL-3), interleukin-6 (IL-6), and granulocyte-macrophage colony-stimulating factor (GM-CSF) as described in the Materials and Methods (“Cell culture” section). Our data revealed a progressive increase in *UBA1-CDK16* expression on days 3 and 5 during the induced myeloid differentiation (Fig. 4B).

Recognizing the significant up-regulation of *UBA1-CDK16* during in vitro myeloid differentiation, we decided to study its function throughout this differentiation process. We designed two short hairpin RNAs (shRNAs) (shUC1 and shUC2) targeting the chimeric RNA junction sequence to specifically reduce *UBA1-CDK16* expression in expanded HSCs through lentivirus transduction (Fig. 4C). Three days after initiating myeloid differentiation, we observed an efficient knockdown of *UBA1-CDK16*, with no changes in the expression of the parental transcripts (Fig. 4D and fig. S7B). Following 5 days of differentiation, flow cytometry analysis revealed a predominant myeloblast subset characterized by the CD33^+^CD11b^+^ population (fig. S7C). Upon *UBA1-CDK16* knockdown, we observed a notable increase in the CD11b^+^ population, indicated by enhanced fluorescence intensity in the female donor (Fig. 4E). In contrast, the male donor, used as an shRNA off-target control, exhibited a CD11b fluorescence distribution similar to that of the short hairpin green fluorescent protein (shGFP) control (Fig. 4E). To further confirm the effect of *UBA1-CDK16* on CD11b^+^ population development and to count for donor variability, we replicated this experiment using two additional pairs of HSC donors. In the second pair, we consistently observed an increase in the CD11b^+^ population resulting from the loss of *UBA1-CDK16* in a female-specific manner (fig. S7D). In the third pair, we observed the same phenotype in both the female and “male” donors, with notable shift in CD11b fluorescence intensity following knockdown with both shRNAs. Notably, the abnormal “male” donor was later found to be positive for *UBA1-CDK16* (fig. S7E). Consistently, RT-qPCR analysis detected elevated *CD11b* mRNA levels in all *UBA1-CDK16*–positive donors, including the abnormal “male” donor (Fig. 4F and fig. S7F).

To comprehensively investigate the impact of reduced *UBA1-CDK16* expression on myeloid-lineage cell distribution, we performed single-cell RNA-seq (scRNA-seq) on an additional female donor. We analyzed immune cell subpopulations following *UBA1-CDK16* knockdown and 3 days of in vitro myeloid differentiation. We observed a notable increase in neutrophils, monocytes, and dendritic cells, accompanied by a decrease in megakaryocytes, platelets, and erythroid cells (Fig. 4G and fig. S7G). Previous studies have shown that antagonistic master regulators, such as GATA-1 and PU.1, play key roles in balancing granulocyte-macrophage versus megakaryocyte-erythroid lineage development (*43*). Our findings strongly suggest this female-specific chimeric RNA, *UBA1-CDK16*, may act as an emerging regulator of hematopoiesis, potentially functioning as a checkpoint to restrain excess myeloid differentiation in females. Recent discoveries have demonstrated the important role of neutrophils in autoimmune disorders and inflammatory responses (*44*). With the observation of increased neutrophils upon loss of *UBA1-CDK16*, the chimeric RNA may represent a distinct protective mechanism in females against excessive autoimmunity.

### Abnormal expression of *UBA1-CDK16* in patients with COVID-19

**Fig. 5.**
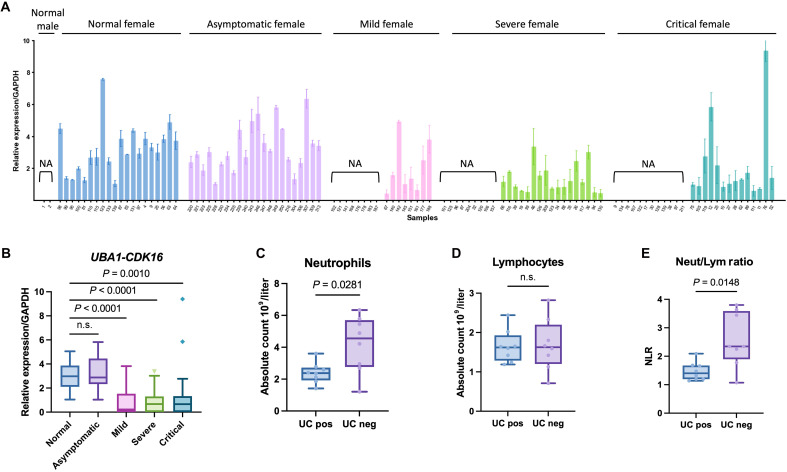
Abnormal expression of *UBA1-CDK16* in female patients with COVID-19. (**A**) RT-qPCR revealed the expression of *UBA1-CDK16* in each female patient with COVID-19. Normal females were used as positive control, while normal males were used as negative control. (**B**) RT-qPCR revealed the expression of *UBA1-CDK16* in female patients with COVID-19 grouped by symptoms, including asymptomatic, mild, severe, and critical cases. (**C** to **E**) Neutrophil and lymphocyte counts and the NLR were evaluated in all female patients with COVID-19 who tested positive or negative for *UBA1-CDK16*. UC pos indicates samples positive for *UBA1-CDK16* expression, and UC neg indicates samples negative for *UBA1-CDK16* expression. n.s., not significant.

During the recent COVID-19 pandemic, multiple studies have highlighted a gender disparity, with males experiencing more severe COVID-19 symptoms and higher mortality rates compared with females. These differences have been linked to sex-biased immune responses, wherein females exhibit a more robust T cell activation, while males demonstrate stronger innate immune responses (*45*). Given our understanding of *UBA1-CDK16*’s potential role in regulating neutrophil-mediated inflammatory responses, we aimed to further investigate its involvement in responding to COVID-19 infection. We first examined the expression levels of *UBA1-CDK16* in female patients with COVID-19 categorized by symptom severity, including asymptomatic, mild, severe, and critically ill or dead. We used whole blood samples from healthy individuals as control, where *UBA1-CDK16* was consistently present in all females and absent in males. Our findings showed that *UBA1-CDK16* was normally expressed in all 22 asymptomatic female patients with COVID-19 (Fig. 5A). However, as the severity of COVID-19 symptoms increased, a subgroup of female patients lost the expression of *UBA1-CDK16* (Fig. 5A)*.* The quantification analysis indicated a marked decrease in the *UBA1-CDK16* expression, which correlated with the increasing severity of symptoms (Fig. 5B). To investigate the *UBA1-CDK16*–correlated sex-biased COVID-19 immune phenotypes, we compared blood test results between female patients who tested positive and negative for *UBA1-CDK16*. We noticed a higher level of neutrophil counts in female patients who had lost *UBA1-CDK16* expression (Fig. 5C). However, no obvious difference was observed in lymphocyte counts (Fig. 5D). Recent studies have reported the association of elevated monocyte counts with the increased COVID-19 severity (*46*) and identified the neutrophil-to-lymphocyte ratio (NLR) as an independent COVID-19 prognosis marker (*47*, *48*). Consistently, we observed a significant elevation of NLR in the symptomatic patients who tested negative for *UBA1-CDK16* (Fig. 5E). To further examine the relationship between *UBA1-CDK16*–associated changes in neutrophil counts and COVID-19 disease progression, we analyzed the blood test results in each symptom group (fig. S8, A to C). An association between loss of *UBA1-CDK16* expression and increased neutrophil counts was observed specifically in the mild-disease group. These findings suggest that *UBA1-CDK16* may exert an inhibitory regulatory effect on neutrophil development or expansion at earlier stages of disease, with potential implications for the progression of COVID-19.

## DISCUSSION

Chimeric RNAs were once thought to be exclusively generated by gene fusion at the DNA level, thus unique to cancer cells (*7*, *9*). Recent studies have demonstrated that these chimeric RNAs can also result from intergenic splicing and be part of normal physiology (*12*, *49*, *50*). In this study, we uncovered an unrecognized sex-specific chimeric RNA, *UBA1-CDK16*, almost exclusively found in blood. This chimeric RNA is the result of cis-SAGe, occurring solely from the inactive X chromosome. As a result, it is expressed in females, while the parental genes are expressed in both males and females. This finding supports the notion that chimeric RNAs may be a means to expand the transcriptome and diversify the pool of encoded genetic information.

The detection of chimeric RNA is highly dependent on the analytical method. Although multiple computational pipelines are available, each differs in sensitivity and specificity. In this study, we opted to use EricScript on the basis of prior comparative evaluations (*33*, *51*). However, a more updated benchmark study has ranked EricScript low because of its high false-positive rate (*52*). In addition, short-read RNA-seq has inherently lower sensitivity than RT-PCR, and its performance is further constrained by read depth, alignment ambiguity of chimeric reads, and algorithm thresholds. To solve the low detection rate of the chimeric RNA through bioinformatic analysis, we therefore conducted comprehensive experimental validation. By using RT-qPCR, the same GTEx blood samples used for RNA-seq analysis exhibited a 100% detection rate in females, confirming the female-specific characteristics of *UBA1-CDK16*. In the clinical samples, the detection rate was ~92%. While this supports the female-specific nature of *UBA1-CDK16*, the outliers in clinical samples reflect the biological variability such as physiological conditions, disease- or treatment-associated effects, or other environmental influences.

In humans, both *UBA1* and *CDK16* escape X inactivation (*53*), which presumably contributes to the transcriptional read-through initiated by the *UBA1* promoter. We have confirmed the previous findings regarding the mechanisms of transcriptional read-through facilitated by chromatin interactions within the 5′ regions of parental genes (*38*). However, it is worth noting that this chromatin loop alone is insufficient for the formation of *UBA1-CDK16*. Our study highlighted the involvement of alternative splicing in cis-SAGe, a process mediated by differences in chromatin structure on the inactive X chromosome (*54*). The distinct chromatin loop formed between the junction splicing sites distinguishes the chromatin structure between the active and inactive chromosomes, ultimately leading to the formation of the chimeric RNA in females.

The evolutionary trajectory of *UBA1-CDK16* across species provides compelling evidence for its functional significance. While we observed its presence in a different isoform in both male and female mice, the emergence of a female-specific isoform in baboons and rhesus monkeys suggests that this chimeric RNA has evolved to play specialized roles. This underscores the importance of further investigating its functions and implications within the evolutionary context of the blood immune system and sexual dimorphism.

The functional study of *UBA1-CDK16* was largely limited by its exclusive expression in primary cells. The chimeric RNA was undetectable in lymphoblastoid cell lines, potentially attributed to Epstein-Barr virus transformation or in vitro culture conditions. Because of this, conventional cell lines proved unsuitable for investigating the functions of *UBA1-CDK16*. To overcome this limitation, we evaluated the chimeric RNA expression levels during myeloid differentiation from primary CD34^+^ cells and observed a significant increase in *UBA1-CDK16* expression. We thus used this experimental system to study the phenotype effects influenced by *UBA1-CDK16* throughout myeloid differentiation.

Sexual dimorphism in hematological systems has been a subject of increasing interest, with evidence pointing toward the involvement of X-linked genetic factors. The X chromosome inactivation escape is likely to contribute to female-specific functions in immune cells (*24*, *55*). A recent study has highlighted the role of *Xist* ribonucleoproteins in promoting female sex-biased autoimmunity (*30*). Our findings here address the other side and illustrate that females have some protective mechanism against excessive autoimmunity. Upon the knockdown of *UBA1-CDK16* in HSCs, followed by myeloid differentiation, we observed an increased population of myeloid cells including neutrophils, monocytes, and dendritic cells, which are key players in autoimmune responses. Although further investigations are needed to fully elucidate the precise mechanisms involved, our study has provided a critical step in uncovering the role of sexual transcriptomic variants in contributing to sex-biased immunity.

Appreciating the function of *UBA1-CDK16* in myeloid differentiation, we aimed to explore its potential implications in sex-biased diseases. Multiple studies have reported sex differences in the immune responses of patients with COVID-19 (*45*, *56*). In our study, we observed higher counts of neutrophils in mild female patients with COVID-19 who tested negative for *UBA1-CDK16*, further confirming the inhibitory role of this chimeric RNA in myeloid differentiation. While a subset of patients in severe and critical groups also tested negative for *UBA1-CDK16*, no difference in neutrophil counts was observed. It is possible that different stages of virus infection may involve distinct immune regulatory mechanisms that are not mediated by *UBA1-CDK16.* The increased NLR, a known prognosis marker (*47*, *48*), observed in mild female patients with COVID-19 who have lost *UBA1-CDK16* expression raises intriguing questions about the functional consequences of *UBA1-CDK16* loss in myeloid cells during infection. Failure to detect *UBA1-CDK16* may be associated with poor outcomes in patients with COVID-19. In addition, the association of *UBA1-CDK16* transcripts with myeloid cell differentiation and inflammation is highly plausible, given that VEXAS syndrome (Vacuoles, E1 enzyme, X-linked, Autoinflammatory, Somatic), a severe adult-onset inflammatory disorder, is caused by somatic mutations in the *UBA1* gene predominantly in myeloid cells (*57*, *58*). Moreover, many immune-related disorders, such as cancer, infectious diseases, or autoimmune diseases, exhibit sex differences, especially in myeloid cells. To further understand the role of *UBA1-CDK16* in disease contexts, future studies will screen for the aberrant expression of *UBA1-CDK16* in a range of sex-biased diseases including VEXAS syndrome. Given its enrichment in blood, this female-specific chimeric RNA may offer valuable insights into its potential use as a diagnostic or prognostic biomarker.

## MATERIALS AND METHODS

### Bioinformatics analyses

Raw RNA-seq datasets (7059) for 44 different human tissues were downloaded from the GTEx Project (GTEx V2, dbGaP Study Accession: phs000424.v2.p1) and subjected to the Next Generation Sequencing Quality Control toolkit (*59*) to filter out low-quality reads. Paired-end sequencing reads were mapped to the human genome (GRCh38/hg38) and analyzed using the software tool EricScript with default parameters (*33*) to identify candidate chimeric RNAs. To remove the putative false positives, chimeric RNAs with an Ericscore (EricScript prediction score) of less than 0.6 were filtered out (*33*). BLAT (*60*) was used to further eliminate putative false-positive chimeric RNAs whose junction sequences have more than 90% identical alignment to existing human transcripts annotated in Ensembl (*61*), RefSeq (*62*), or GENCODE (*63*). Using the junction coordinates, each chimeric RNA was categorized according to the location of breakpoints: E/E (both parental gene breakpoints located at the exon boundaries), E/M (5′ breakpoint at an exon boundary and 3′ breakpoint in the middle exon), M/E (5′ breakpoint in the middle of exon and 3′ gene at an exon boundary), or M/M (both breakpoints located in the middle of exons). Given the high false-positive rate of “M/M” chimeric RNAs, we filtered out them, requiring at least one side falls on canonical exon boundary. The cut of 100 kb was used to define intrachromosomal chimeric RNA category. The occurrence and frequencies of candidate chimeric RNAs were then correlated with the sex of the donors. Pearson’s chi-squared tests were performed between observed fusion frequencies and expected sample frequencies in male and female samples to generate a bubble plot. In this plot, data points are replaced with bubbles, *x* axis plots the distribution of chimeric RNAs, and the *y* axis plots the negative value of the log(base10)-transformed *P* value obtained via chi-squared tests, with the size of each bubble representing the total frequency of each chimera in GTEx blood samples, and the color of each bubble representing the percentage of chimeras observed in female samples. The cutoff line indicates the log-base10 of 0.05, which is 1.3.

### scRNA-seq analyses

Raw sequencing reads were processed using kb-python (version 0.28.2) (*64*) for transcript quantification. The reference index was built with kb ref. using the GRCh38 primary assembly genome and ensembl v112 annotation. Pseudoalignment and quantification were performed with kb count to generate filtered count matrices. Count matrices were imported into R using Seurat (*65*). For each sample, matrices were read with ReadMtx and Seurat objects were created, applying initial filtering to exclude genes detected in fewer than three cells and cells with fewer than 200 detected genes. The samples were merged into a single Seurat object for downstream analysis. Quality control metrics included filtering out cells with fewer than 350 detected genes, fewer than 500 total counts, or greater than 15% mitochondrial gene content. Normalization, variance stabilization, and feature selection were performed using SCTransform, regressing out mitochondrial content. Dimensionality reduction was performed with principal components analysis (50 components) followed by uniform manifold approximation and projection (UMAP) (using the first 30 principal components). Shared nearest neighbor graph construction and Louvain clustering were performed at multiple resolutions, with resolution 0.3 selected for downstream analyses. Cluster identities were visualized using UMAP plots.

To identify cluster-specific marker genes, we used the Seurat v4 workflow with SCTransform normalization. Before marker detection using the DESeq2 method, we applied the PrepSCTFindMarkers function to prepare the SCTransform (SCT) assay data for differential expression analysis. Marker gene identification was performed using FindAllMarkers on the integrated and normalized Seurat object, with the following parameters: minimum log fold change threshold of 0.25, minimum percentage expression cutoff of 0.1, and restricting to positive markers only. Top marker genes for each cluster were selected on the basis of the highest average log fold change. Clusters were annotated on the basis of the marker gene expression and literature.

### Clinical sample collection

The use of human clinical blood samples was approved by the institutional review board (IRB) committee of the University of Virginia (protocol no. 13310). Blood samples obtained from the Department of Pathology at the University of Virginia were deidentified. Buffy coats were separated from a total of 1351 whole blood samples using 2 ml of blood collected in sodium citrate buffer for centrifugation at 4°C and 3000*g* for 15 min.

The COVID-19 study was reviewed and approved by the Institutional Review Board of Tongji Hospital, Tongji Medical College, Huazhong University of Science and Technology, China (TJ-IRB20200405). All enrolled patients provided signed informed consent, and all blood samples were collected for the rest of the standard diagnostic tests, with no additional burden to the patients. Blood samples from 90 patients with COVID-19 without selected comorbidities were collected from Tongji Hospital and Union Hospital of Huazhong University of Science and Technology, Xiangyang Central Hospital, Hubei University of Arts and Science, and Hubei Dazhong Hospital of Chinese Traditional Medicine between 19 February and 26 April 2020. The mean age of patients was 46.7 years old (SD = 13.5). All patients were diagnosed following the guidelines for COVID-19 diagnosis and treatment released by the National Health Commission of the People’s Republic of China. The patients were classified into four groups according to disease severity: i.e., critical, severe, mild, and asymptomatic. The definition criteria for disease severity were described by Wu *et al.* (*66*). The ethylenediaminetetraacetic acid disodium salt (EDTA-2Na)–anticoagulated venous blood samples were separated by centrifugation at 3000 rpm for 7 min at room temperature after standard diagnostic tests.

### RNA extraction

RNA was extracted from buffy coats with TRIzol reagent (Invitrogen) following the manufacturer’s instructions. RNA samples were examined for quantity and quality using NanoDrop (Thermo Fisher Scientific) and reverse transcribed via random primers using the Verso cDNA synthesis kit (Thermo Fisher Scientific). All 1252 clinical blood samples passed quality control, with optical density ratios of 260 nm/280 nm and 260 nm/230 nm, falling within 1.6 to 2.0, and levels of an internal control glyceraldehyde-3-phosphate dehydrogenase (*GAPDH*) within 2 SDs of the mean value.

### RT-PCR and Sanger sequencing

Primer pairs for PCR were designed using Primer3 (Whitehead Institute for Biomedical Research). A Step One Plus Real-Time PCR System (Applied Biosystems) was used to perform SYBR Green based RT-qPCR assay. Relative RNA levels were calculated using the ΔΔCt method. Gene expression was normalized to that of the housekeeping gene, *GAPDH*. Amplified products were separated using agarose gel electrophoresis. Proper size product bands were purified using the PureLink Quick Gel Extraction Kit (Invitrogen) and sent for Sanger sequencing (Genewiz). Primer sequences and their corresponding genomic coordinates are listed in table S2. Genomic positions were annotated using the following reference genome assemblies: Human (GRCh38/hg38), Mouse (GRCm39/mm39), Marmoset (May 2020; *Callithrix jacchus* cj1700_1.1/calJac4), Baboon (April 2017; Panu_3.0/papAnu4), and Rhesus macaque (February 2019; Mmul_10/rheMac10).

### Chromosome conformation capture

3C was performed as described by Cope and Fraser (*67*). Human PBMCs were isolated by Ficoll-Paque (GE Healthcare) density centrifugation from buffy coats (American Red Cross) and stored in liquid nitrogen. After thawing, 10 million PBMCs were fixed in 1.5% formaldehyde for 10 min. Cross-linking was quenched with 0.25 mM of glycine for 10 min at room temperature, followed by two washes with phosphate-buffered saline (PBS). Cells were resuspended in 10 ml of lysis buffer [50 mM tris–HCl (pH 8), 150 mM NaCl, 0.5% NP-40, 1% Triton X-100, protease inhibitor cocktail (Sigma-Aldrich)] for 30 min on ice. Cell lysates were centrifuged at 2000 rpm for 5 min at 4°C to isolate the nuclei. Nuclei were then resuspended in 500 μl of 1× NEBuffer3 (NEB) containing 0.3% SDS and incubated for 1 hour at 37°C, followed by 1 hour of incubation after adding 10% Triton X-100 (final concentration 1.8%). Nuclei were digested using 800 U of Bam HI (for 5′-5′ loop) or Bgl II (for junction loop) (NEB) on a shaker overnight at 37°C. Digestion was quenched by adding 10% SDS (final concentration 1.6%) and incubated for 25 min at 65°C. The digested nuclei were washed and resuspended by 1× T4 ligase buffer (NEB). Nuclei were then ligated for 4 hours at room temperature by adding 1% Triton X-100, 1 mM adenosine triphosphate, and 4000 U of T4 DNA ligase (NEB). The ligation reaction was stopped by adding 0.5 M EDTA (final concentration 10 mM). The ligated chromatin was followed by proteinase K (final concentration 100 μg/ml) digestion at 65°C overnight and then treated with ribonuclease A (RNase A) (0.5 ug/ml) for 1 hour at 37°C water bath. The DNA was extracted by phenol-chloroform and chloroform and precipitated by 100% ethanol and sodium acetate. DNA pellets were then washed by 70% ethanol and resuspended in 200 μl of H_2_O. 3C DNA (2 μl) was used for qPCR. Unidirectional primers were used to detect ligation interaction frequency. 3C qPCR analysis was performed as described by Naumova *et al.* (*68*). Primes efficiency was normalized by BAC library, and the interaction frequency was normalized against anchor site ligation frequency. Primers for each digestion site and their corresponding genomic coordinates are listed in table S3.

### Chromatin immunoprecipitation

A total of 10 million PBMCs were fixed in 1.5% formaldehyde for 10 min. Cross-linking was quenched with 0.25 mM glycine for 10 min at room temperature, followed by two washes with PBS. Cells were resuspended in 1 ml of lysis buffer [5 mM Pipes (pH 8), 85 mM KCl, 0.5% NP-40, and protease inhibitor cocktail (Sigma-Aldrich)] for 10 min on ice. Cell lysates were centrifuged at 5000 rpm for 5 min at 4°C, and the nuclei were incubated in the nuclei lysis buffer [50 mM tris-HCl (pH 8), 10 mM EDTA, and 1% SDS] for 10 min on ice. Nuclei lysates were sonicated with 30 s on and 30 s off for 20 min in total. Following sonication, nuclei were cleared by centrifugation and subjected to preclearance by incubating with Protein A agarose beads (Cell Signaling). Precleared cell lysates were separately incubated with CTCF or nonspecific immunoglobulin G (IgG) antibodies (Cell Signaling) overnight, followed by incubation with Protein A agarose beads for 2 hours and 4°C. Protein-bound agarose beads were washed sequentially with low-salt wash buffer, high-salt wash buffer, LiCl wash buffer, and Tris-EDTA (TE) buffer. Bead-bound complexes were subjected to elution by IP elution buffer (1% SDS and 0.1 M NaHCO_3_). Eluted complexes were decross-linked by 5 M NaCl overnight at 67°C. All elutes and inputs were degraded in protein/RNA degradation buffer [EDTA, tris-HCl (pH 6.5), proteinase K, and RNase A]. The DNA was extracted by phenol-chloroform and chloroform and precipitated by 100% ethanol and sodium acetate. DNA pellets were then washed by 70% ethanol and resuspended in 60 μl of H_2_O. ChIP DNA (2 μl) was used for qPCR. The percentage of input for each IP or IgG was calculated. Primers for each CTCF binding site and their corresponding genomic coordinates are listed in table S4.

### CRISPR-mediated reorganization of chromatin loop structure

CLOuD9 was performed as described by Morgan *et al.* (*39*). Briefly, guide RNAs (gRNAs) targeting each desired anchor site were designed and cloned into *Staphylococcus aureus* and *Streptococcus pyogenes* constructs. gRNA sequences are listed in table S5. The plasmids were transfected into HEK293T cells through GeneCellin (Bulldog Bio) following manufacturer’s protocol. Forty-eight hours after transfection, cells were selected in growth medium with puromycin (1 μg/ml) and hygromycin (25 μg/ml). Cells were kept in selection media for at least 1 week and were maintained in selection media for the duration of downstream experiments. For dimerization treatment of CLOuD9-transduced cells, 1 mM ABA (Thermo Fisher Scientific) [or an equivalent volume of dimethyl sulfoxide (DMSO) for controls] was added to the culture medium. Medium was changed with fresh ABA or DMSO daily. After a 3-day treatment, cells were collected by trypsin digestion, followed by either 3C analysis or RNA extraction.

### Cell culture

HEK293T cells were maintained in Dulbecco’s modified Eagle’s medium (DMEM) (Gibco), supplemented with 10% fetal bovine serum (VWR), and 1% Pen/Strep solution (Gibco). Cells were cultured at 37°C in 5% CO_2_ humidity.

Cryopreserved CD34^+^ primary human progenitors were purchased from FredHutch and thawed following their instructions. Samples were requested from healthy adult donors aged between 30 and 50 years. CD34^+^ cells were maintained in Iscove modified Dulbecco medium (IMDM) (Gibco), supplemented with 20% BITS 9500 (StemCell Technologies), 2 mM l-glutamine (Gibco), 1% Pen/Strep solution (Gibco), and 1-thioglycerol (Sigma-Aldrich). Undifferentiated CD34^+^ cells were expanded for 4 days in prestimulation medium by adding SCF (100 ng/ml) (PeproTech), FLT3-ligand (100 ng/ml) (PeproTech), human thrombopoietin (TPO) (100 ng/ml) (PeproTech), and IL-3 (20 ng/ml) (PeproTech). For myeloid differentiation, the expanded CD34^+^ cells were cultured in basal medium with GM-CSF (20 ng/μl) (BioLegend) and 1× CC100 (StemCell Technologies) consisting of cytokines SCF, FLt3-ligand, IL-3, and IL-6.

### shRNA transduction

Oligo DNA sequence of shUC1 and shUC2 were cloned into pLKO.1 plasmid vector. shRNA sequences are listed in table S5. The plasmid was packaged into lentivirus with packaging constructs pCMV and pMD6.G. Briefly, 750,000 HEK293T cells per well were seeded into a six-well plate. Twenty-four hours after seeding, plasmids were transfected into HEK293T cells using GeneCellin (Bulldog Bio) following the manufacturer’s protocol. After 24 hours, the media was changed to viral production media Opti-MEM (Gibco). After 24 hours, viral production media was collected and spun at 500*g* for 5 min to remove cell debris. RetroNectin (Takara) was used to coat six-well plate following the manufacturer’s protocol. For transduction, 1 ml of viral supernatant was preloaded to the RetroNectin-coated plate and incubated at room temperature for 30 min. The viral supernatant was discarded before adding cells. A total of 500,000 prestimulated CD34^+^ cells were suspended in 1 ml of viral supernatant and 1 ml of prestimulation medium with protamine sulfate (5 μg/ml) (Sigma-Aldrich). The cell mixture was added to the RetroNectin-coated plate and incubated at 37°C in 5% CO_2_ humidity for 2 hours. The plate was then spun at 2000 rpm for 90 min and incubated at 37°C in 5% CO_2_ humidity overnight. After 24 hours, cells were changed with 1 ml of fresh prestimulation medium and 1 ml of viral supernatant. After 24 hours, cells were selected in prestimulation medium with puromycin (1.5 μg/ml) for 3 days. Successfully transduced cells were selected and initiated with myeloid differentiation.

### Cell sorting

A total of 20 million PBMCs were washed in RPMI 1640 media (Gibco) twice before being resuspended in 1 ml of sterile PBS with 5% fetal bovine serum (FBS) in a sterile fluorescence-activated cell sorting (FACS) tube. Antibodies were added to stain surface markers for 30 min at 4°C (CD3 phycoerythrin (PE), Life Technologies; CD8 fluorescein isothiocyanate (FITC), Life Technologies; CD11b AF700, Life Technologies CD19 PerCP-Cy5.5, BioLegend; C33 BV605, BioLegend; CD56 BV711, BD). After 30 min, cells were washed twice with PBS + 5% FBS and then resuspended in buffer with 4′,6-diamidino-2-phenylindole (1:25,000, Sigma-Aldrich) for FACS sorting. An Influx Cell Sorter (Becton Dickinson) was used to sort cells into subsets, using PBS with 20% FBS as the collection buffer.

### Flow cytometry

Myeloid differentiated cells were harvested on day 3 and day 5. Cells were washed in PBS and resuspended in 100 μl of PBS + 5% FBS. Antibodies were added to stain surface markers for 30 min at 4°C (CD33 BV711, BD; CD11b APC-Cy7, BD; Zombie Violet, BioLegend). After 30 min, cells were washed with PBS + 5% FBS and fixed in 16% paraformaldehyde. Cells were washed and resuspended in 400 μl of PBS + 5% FBS. An Attune flow cytometer (Thermo Fisher Scientific) was used to acquire data. Zombie Violet was used to gate out live cells. All live cells were gated out on the basis of their forward scatter area (FSC-A) and side scatter area (SSC-A) profile. Single cells were then gated out with FSC-A versus FSC-H and used for FACS analysis. The analysis was performed using FCS Express software.

### Statistics

All quantitative data are presented as the means ± SD. We used unpaired two-tailed *t* tests or one-way analyses of variance (ANOVAs) for variable analysis. *P* values < 0.05 were considered statistically significant.
