# Supplementary material for: UBA1-CDK16: A female-specific chimeric RNA emerging through evolution and involved in immune regulation
Source: Sci Adv. 2026 May 29;12(22):eadz9784. doi: 10.1126/sciadv.adz9784 (PMC13220885; doi:10.1126/sciadv.adz9784)
Supplement: Supplementary file 1 — Figs. S1 to S8 Legends for tables S1 to S5 [file sciadv.adz9784_sm.pdf]

Science Advances
AAAS

Supplementary Materials for

UBA1-CDK16: A female-specific chimeric RNA emerging through evolution and involved in immune regulation

Xinrui Shi *et al.*

Corresponding author: Hui Li, hl9r@virginia.edu

Sci. Adv. **12**, eadz9784 (2026)
DOI: 10.1126/sciadv.adz9784

The PDF file includes:

Figs. S1 to S8
Legends for tables S1 to S5

Other Supplementary Material for this manuscript includes the following:

Tables S1 to S5

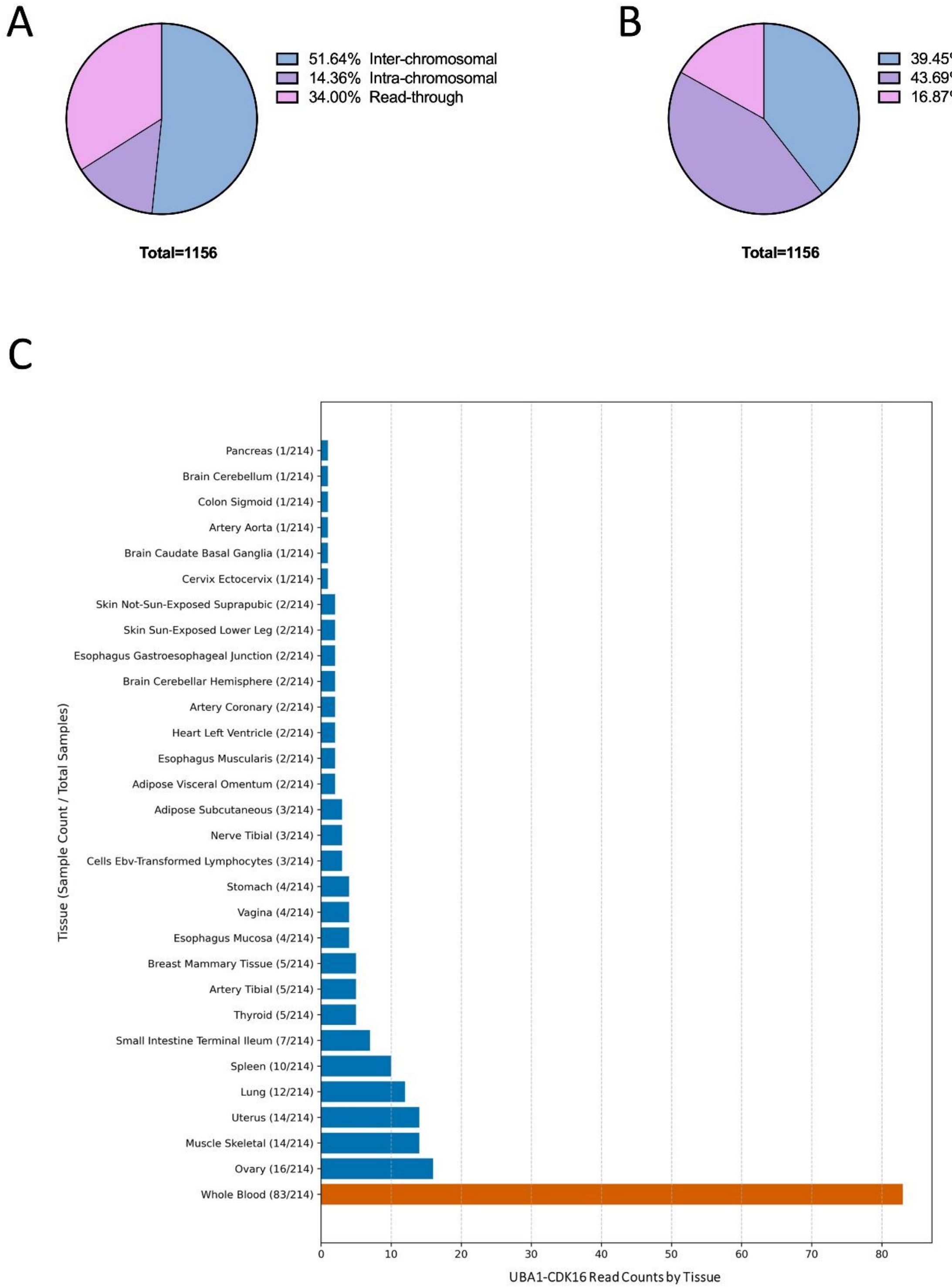

Fig. S1. Distribution of recurrent chimeric RNAs identified in GTEx whole blood RNA-seq data. (A) Types of chimeric RNAs based on parental gene location. (B) Different EM categories based on junction site location. (C) Distribution of *UBA1-CDK16* read counts detected by EricScript across female tissues in the GTEx RNA-seq dataset.

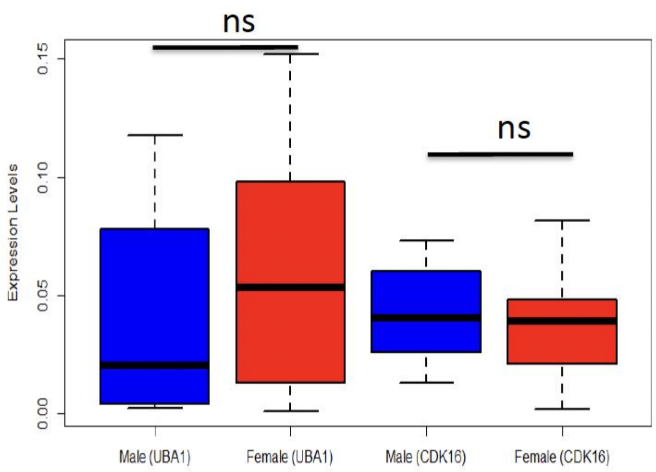

Fig. S2. ***UBA1* and *CDK16* genes expression in males and females.** RT-qPCR detection of parental genes in 15 buffy coat samples. The transcripts were normalized to an internal control *GAPDH.* No statistically significant difference was observed.

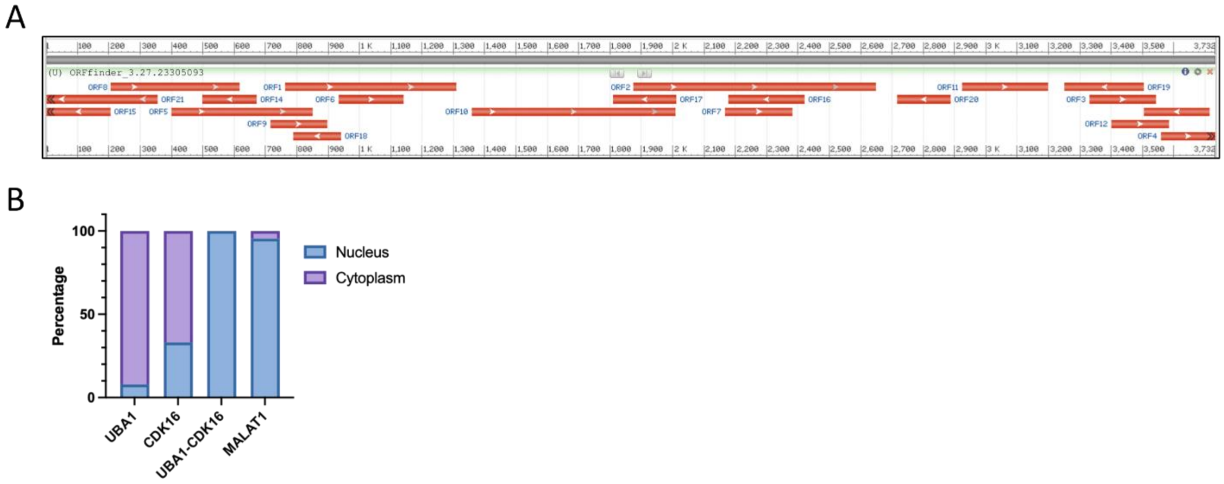

Fig. S3. ***UBA1-CDK16* is a long non-coding chimeric RNA.** (A) The ORFfinder output from NCBI indicated all possible open reading frames (ORFs) within the full-length sequence of *UBA1-CDK16*. The output only displays ORFs that start with ATG, with the minimal ORF length being 150 nucleotides. (B) RT-qPCR detection of nuclear and cytoplasmic RNA levels for *UBA1-CDK16* and its parental genes. The long non-coding RNA *MALAT1,* known for its enrichment in the nucleus, was used as a positive control.

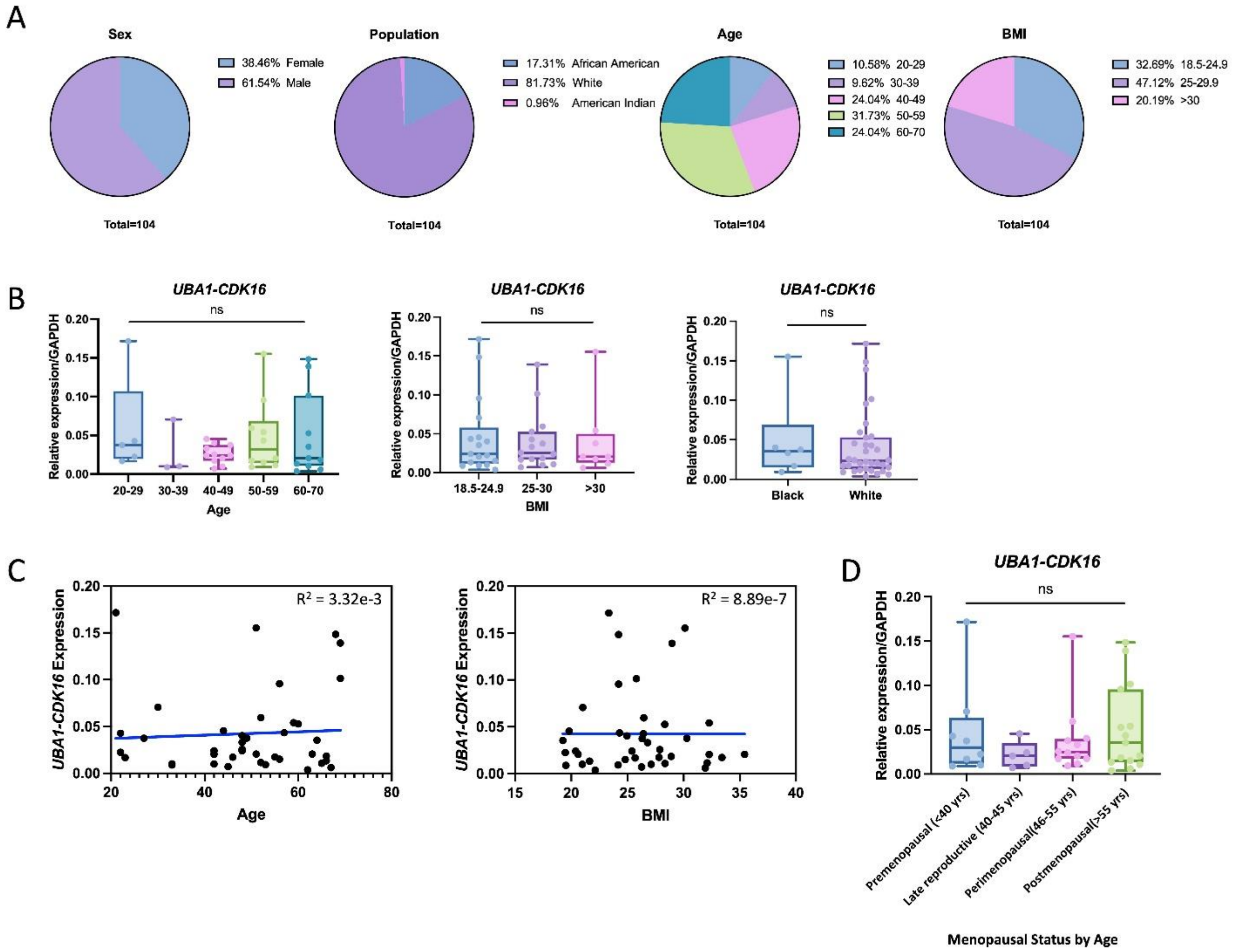

Fig. S4. ***UBA-CDK16*** **expression in GTEx blood samples.** (A) Distribution of samples in sex, population, age, and BMI. (B) RT-qPCR detection of *UBA1-CDK16* expressions in different ages, BMI, or population groups. (C) Linear regression model of *UBA1-CDK16* expression with age or BMI. R^2 indicated the non-significant correlation. (D) RT-qPCR detection of *UBA1-CDK16* expression across menopausal status groups. Transcript levels were normalized to the internal control *GAPDH.* No statistically significant differences were observed among groups.

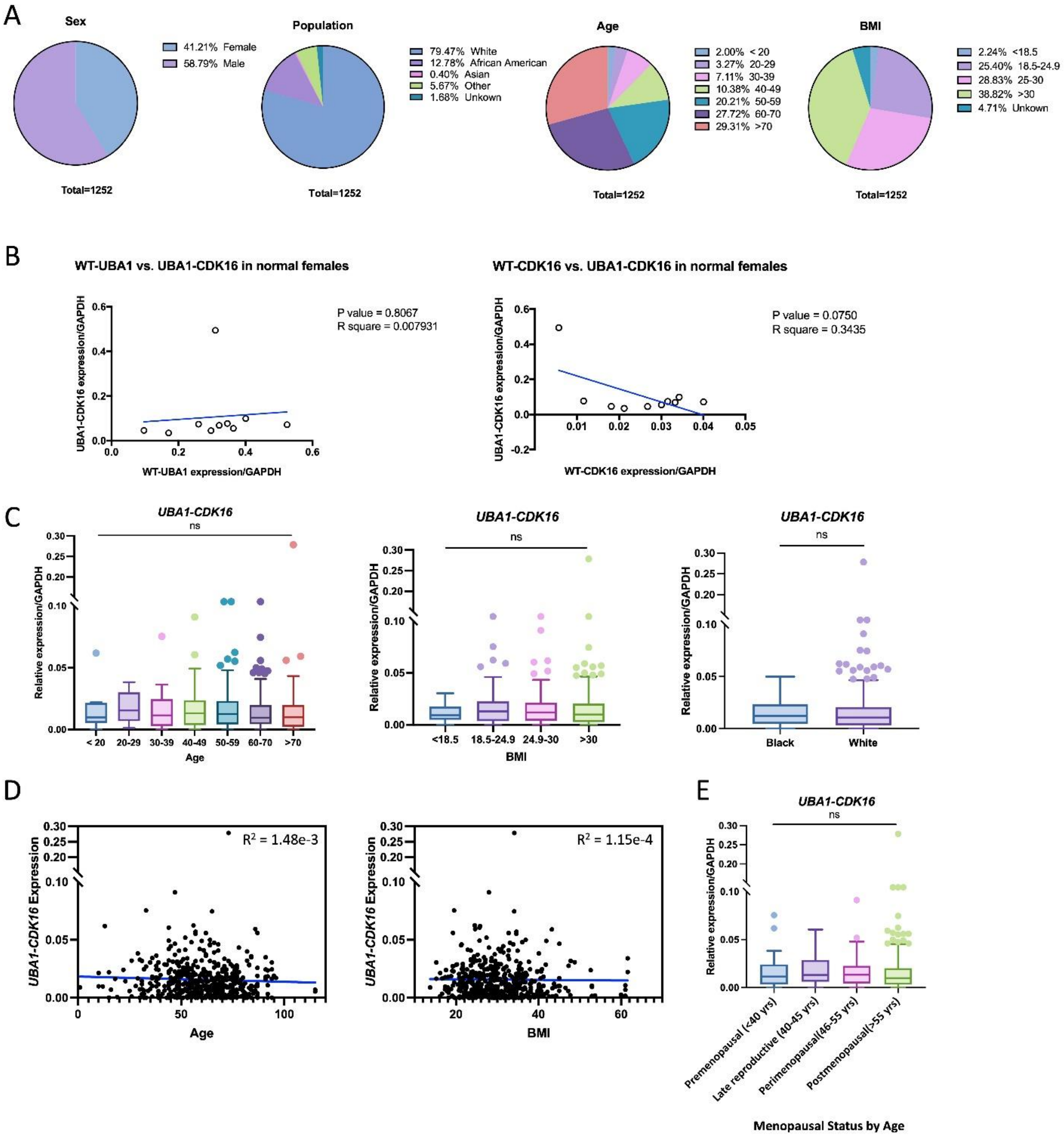

Fig. S5. *UBA-CDK16* expression in clinical blood samples. (A) Distribution of clinical samples in sex, population, age, and BMI. (B) Linear regression model of *UBA1-CDK16* expression with its parental genes *UBA1* or *CDK16* expression. Expression level was measured by RT-qPCR and normalized against *GAPDH*. P values and R^2 values indicated the non-significant correlation. (C) RT-qPCR detection of *UBA1-CDK16* expression in different age, BMI, or population groups. (D) Linear regression model of *UBA1-CDK16* expression with age or BMI. R^2 indicated the non-significant correlation. (E) RT-qPCR detection of *UBA1-CDK16* expression across menopausal status groups. Transcript levels were normalized to the internal control *GAPDH.* No statistically significant differences were observed among groups.

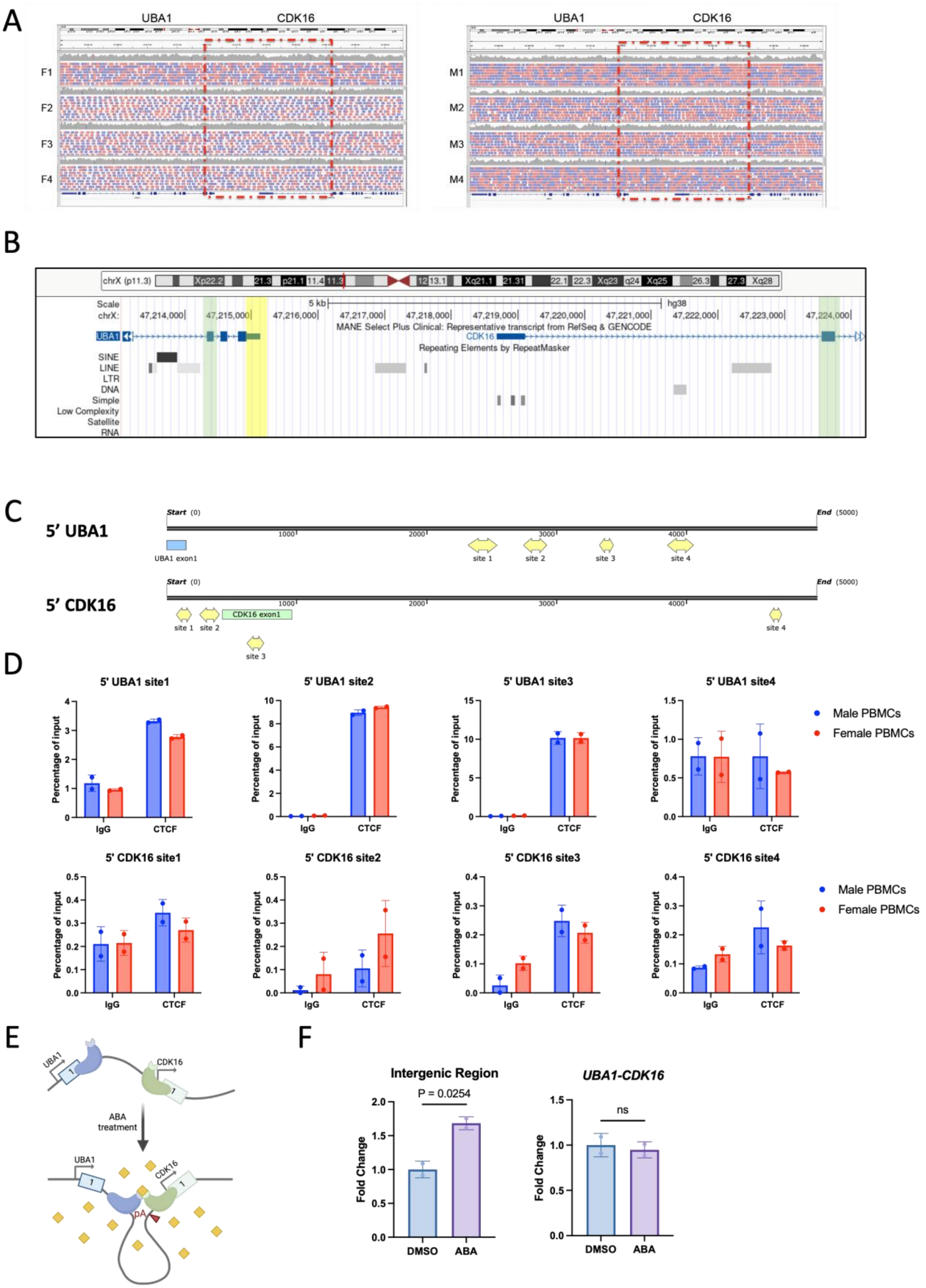
A
UBA1
CDK16
F1
F2
F3
F4
M1
M2
M3
M4
B
chrX (p11.3)
Scale
chrX:
5 kb
hg38
MANE Select Plus Clinical: Representative transcript from RefSeq & GENCODE
UBA1
CDK16
Repeating Elements by RepeatMasker
SINE
LINE
LTR
DNA
Simple
Low Complexity
Satellite
RNA
C
5' UBA1
5' CDK16
Start (0)
End (5000)
UBA1 exon1
CDK16 exon1
site 1
site 2
site 3
site 4
D
5' UBA1 site1
5' UBA1 site2
5' UBA1 site3
5' UBA1 site4
5' CDK16 site1
5' CDK16 site2
5' CDK16 site3
5' CDK16 site4
Percentage of input
IgG
CTCF
Male PBMCs
Female PBMCs
E
UBA1
CDK16
ABA treatment
F
Intergenic Region
P = 0.0254
UBA1-CDK16
ns
Fold Change
DMSO
ABA

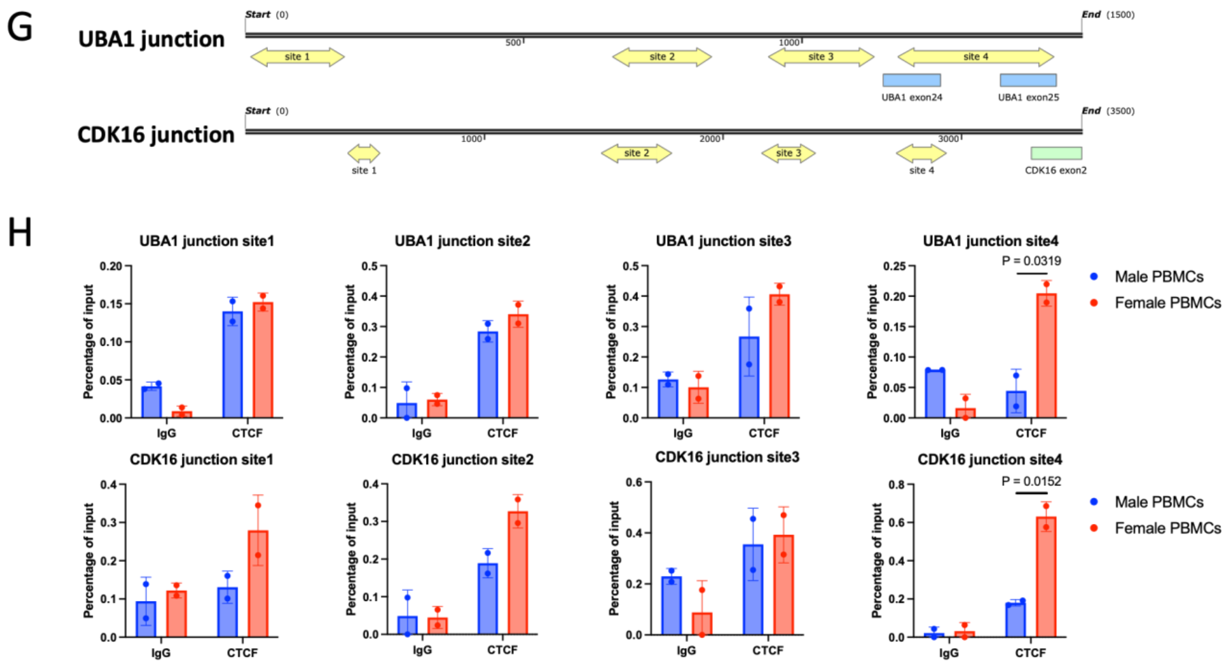

Fig. S6. *UBA1-CDK16* is a product of cis-SAGe through alternative splicing. (A) Whole genome sequencing of GTEx whole blood samples showed no evidence of interstitial deletion between *UBA1* and *CDK16* in four females and four males. (B) RepeatMasker tracks along *UBA1-CDK16* locus are shown in the UCSC Genome Browser (hg38). The transcription termination site of *UBA1* is highlighted in yellow, and the junction sites of *UBA1-CDK16* are highlighted in green. (C) Schematic of CTCF binding sites at 5' regions of *UBA1* and *CDK16*. CTCF binding sites were predicted by the CTCFBSDB 2.0 database. (D) CTCF ChIP-qPCR at predicted CTCF binding sites in male and female PBMCs. IgG antibody was used as control. n = 2 biological replicates. (E) CLOuD9 experimental design with sgRNAs targeting the 5' regions of *UBA1* and *CDK16*. Addition of abscisic acid (ABA, yellow) brings two complementary CLOuD9 constructs (blue and green) into proximity, inducing 5'-5' chromatin loop. (F) cis-SAGe-qPCR revealed a significant increase in precursor readthrough mRNA after ABA treatment. The relative expression level was normalized against *CDK16* using primers targeting exon 1, and fold change was normalized against samples treated with DMSO. No statistically significant change was detected on mature chimeric RNA *UBA1-CDK16* revealed by RT-qPCR. n = 2 biological replicates. (G) Schematic of CTCF binding sites at junction regions of *UBA1* and *CDK16*. CTCF binding sites were predicted by the CTCFBSDB 2.0 database. (H) CTCF ChIP-qPCR at predicted CTCF binding sites in male and female PBMCs. IgG antibody was used as control. n = 2 biological replicates.

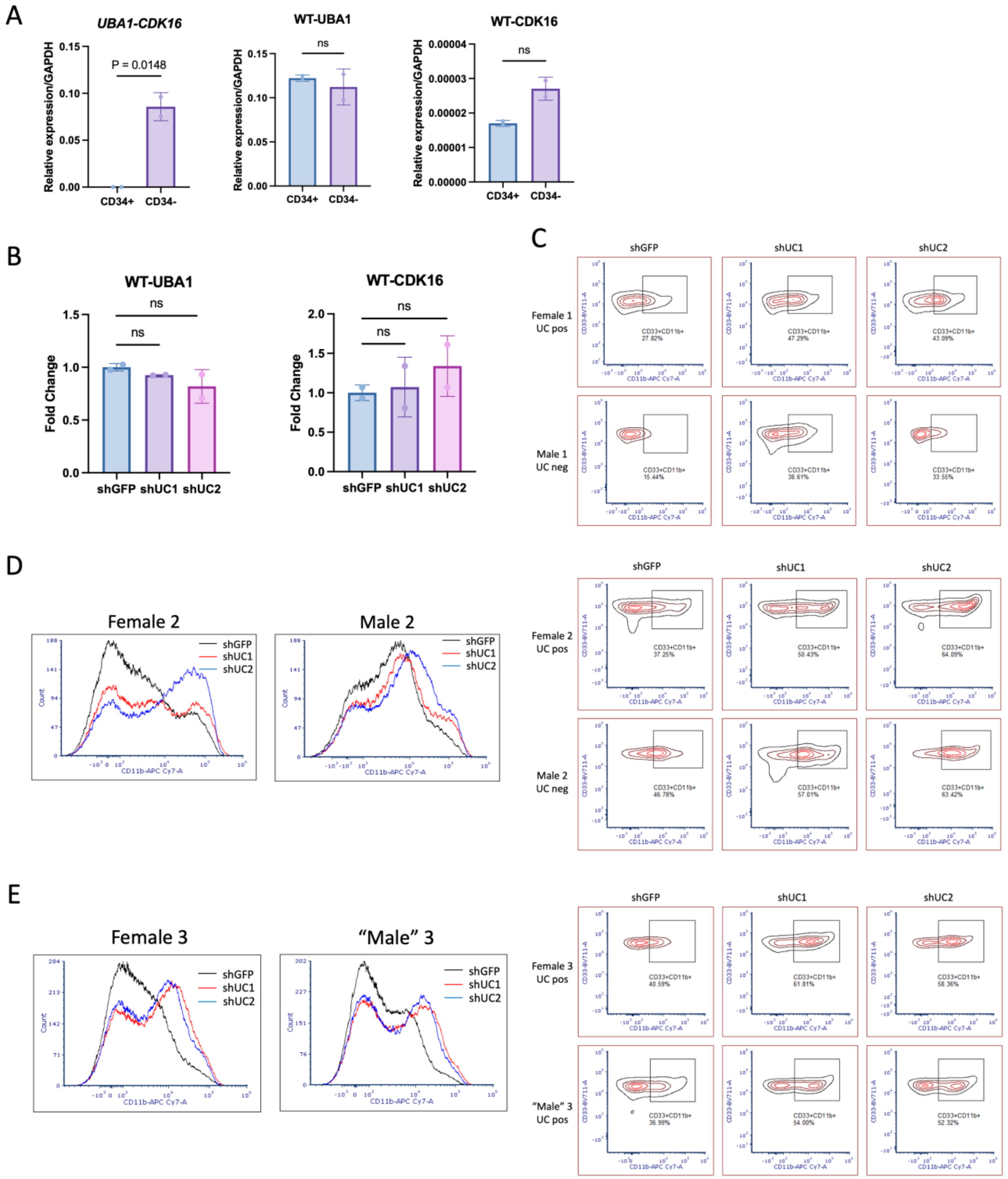
A
UBA1-CDK16
P = 0.0148
Relative expression/GAPDH
CD34+
CD34-
WT-UBA1
ns
WT-CDK16
B
WT-UBA1
WT-CDK16
Fold Change
shGFP
shUC1
shUC2
C
Female 1
UC pos
Male 1
UC neg
CD33+CD11b+
27.82%
47.29%
43.09%
15.44%
38.61%
33.55%
CD33-BV711-A
CD11b-APC Cy7-A
D
Female 2
Male 2
Count
UC pos
UC neg
37.25%
50.43%
64.09%
46.78%
57.01%
63.42%
E
Female 3
"Male" 3
40.59%
61.81%
58.36%
36.99%
54.00%
52.32%

F

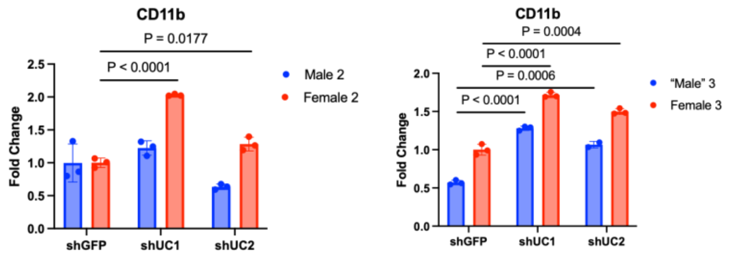

G

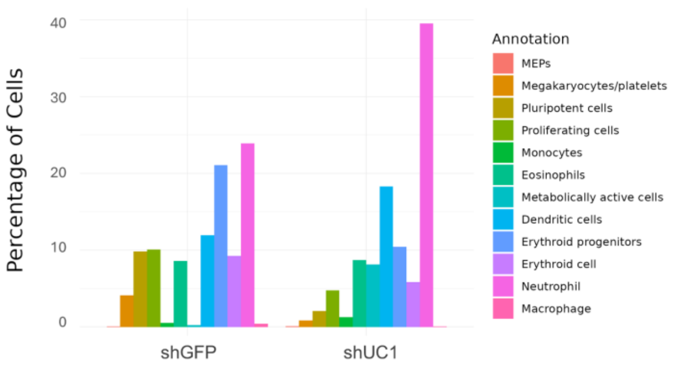

Fig. S7. ***UBA1-CDK16* inhibits CD11b expression during myeloid differentiation.** (A) RT-qPCR detection of chimeric RNA *UBA1-CDK16* and its parental genes expression in CD34$^+$ and CD34$^-$ cells. CD34$^+$ cells were isolated from PBMCs using CD34 microbeads, while CD34$^-$ cells comprised all remaining cells. A statistically significant difference was observed in chimeric RNA expression between CD34$^+$ and CD34$^-$ cells. n = 2 biological replicates. (B) Parental genes expression upon *UBA1-CDK16* knockdown by shUC1 and shUC2. shGFP was used as negative control. n = 2 biological replicates. (C) Flow cytometry analysis of single cells gated for CD33$^+$CD11b$^+$ myeloblasts in CD34$^+$ cells undergo myeloid differentiation upon *UBA1-CDK16* knockdown. UC pos indicates samples positive for *UBA1–CDK16* expression and UC neg indicates samples negative for *UBA1–CDK16* expression. (D, E) The fluorescent intensity distribution of CD11b and flow cytometry analysis of single cells gated for CD33$^+$CD11b$^+$ myeloblasts in the other two pairs of CD34$^+$ cells. (F) *CD11b* mRNA expression detected by RT-qPCR in the other two pairs of CD34$^+$ cells. n = 3 biological replicates. (G) Distribution of identified blood cell types compared between shUC1 and shGFP samples from single cell RNA-Seq. MEPs represent megakaryocyte-erythroid progenitors.

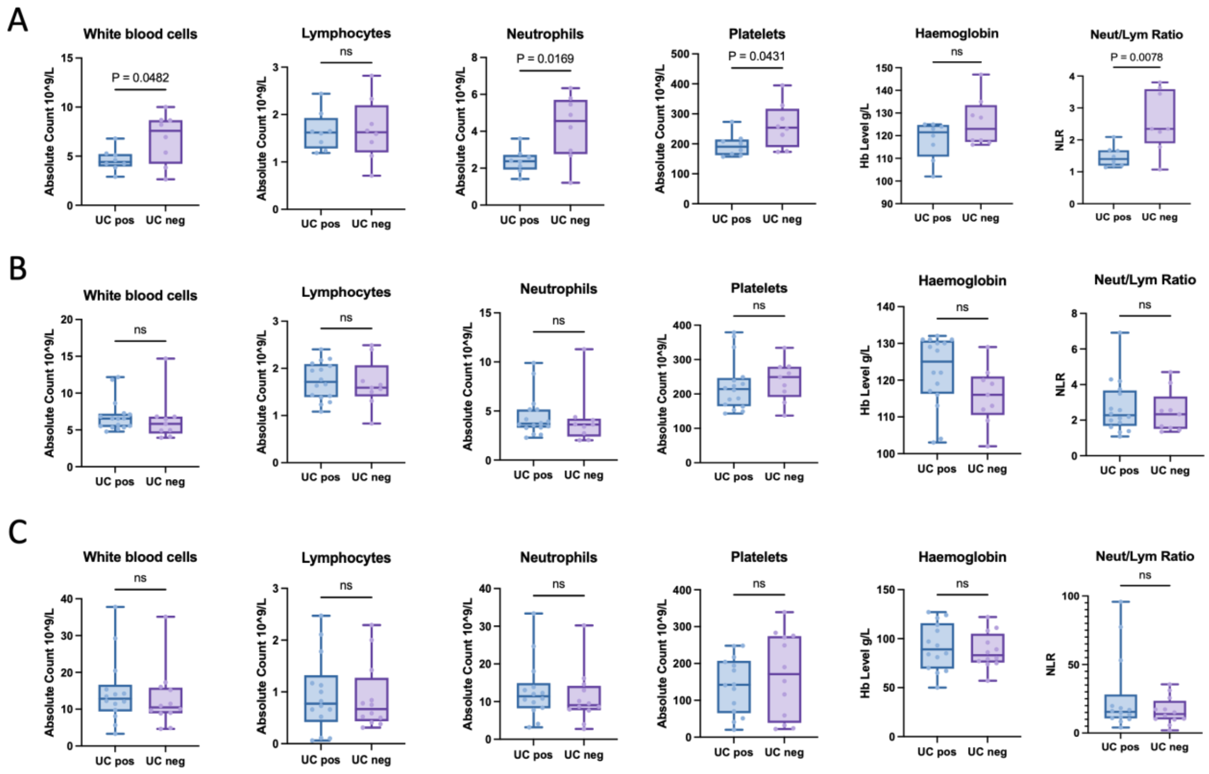

Fig. S8. Blood cell counts in COVID-19 female patients. White blood cells, Lymphocytes, Neutrophils, Platelets counts, Hemoglobin level, and NLR were detected in (A) mild, (B) severe, and (C) critical COVID-19 female patients. Comparison was made between patients who detected positive or negative for *UBA1-CDK16.*

List of supplementary excel files:

Table S1. List of recurrent chimeric RNAs identified in whole blood RNA-seq data

Table S2. PCR primer sequences

Table S3. 3C primer sequences

Table S4. CTCF-ChIP primer sequences

Table S5. shRNA and sgRNA sequences
